# The basic reproductive number and particle-to-plaque ratio: comparison of these two parameters of viral infectivity

**DOI:** 10.1186/s12985-021-01566-4

**Published:** 2021-04-30

**Authors:** Winston McCormick, Leonard A. Mermel

**Affiliations:** 1 Warren Alpert Medical School of Brown University, 222 Richmond St, Providence, RI 02903 USA; 2 Department of Medicine, Warren Alpert Medical School of Brown University, 222 Richmond St, Providence, RI 02903 USA; 3 Department of Epidemiology & Infection Control, and Division of Infectious Diseases, Rhode Island Hospital, 593 Eddy St, Providence, RI 02903 USA

**Keywords:** Basic reproductive number, Particle-to-plaque, Particle-to-pfu, COVID-19, Viral transmission

## Abstract

The COVID-19 pandemic has brought more widespread attention to the basic reproductive number (R_o_), an epidemiologic measurement. A lesser-known measure of virologic infectivity is the particle-to-plaque ratio (P:PFU). We suggest that comparison between the two parameters may assist in better understanding viral transmission dynamics.

As the COVID-19 pandemic continues, attention has been brought to the epidemiologic measure known as the basic reproductive number (R_o_), the expected number of cases arising from an index case in a susceptible population [1–5]. The R_o_ is differentiated from R_e_ or R_t_, the effective reproduction number, which accounts for public health measures such as vaccination, contact tracing, or social distancing [2, 5, 6]. The R_o_ indicates the potential for viral transmission in a population. When R_o_ > 1, the virus exhibits spread within a population, and when it is less than 1, it does not have the potential to spread. The R_o_ is determined from mathematical models and must be interpreted under the context that models are often imperfect. Indeed, to be a true reflection of the R_o_, the model cannot involve any public health measures taken to delay viral transmission. A major limitation of R_o_ is that it is difficult to compare R_o_ values of two viruses if they are calculated using different models. Although there are many models to calculate R_o,_ a SEIR compartmental method is among the simplest and widely used available methods [7]. The higher the R_o_, the more public health measures must be expended to bring the R_e_ < 1 needed for an epidemic or pandemic to cease [2, 8]. The R_o_ can be manipulated to indicate parameters vital to control measures. For example, ${R}_{o}^{-1}$ is the endemic equilibrium proportion of the population that will remain susceptible, and $1-\frac{1}{{R}_{o}}$ alerts public health officials to the proportion of a population that must be immunized to acquire herd immunity [1]. Imprecision in determining R_o_ can lead to public health measures that are either too relaxed or too strenuous, leading to spread that is not adequately controlled or burnout among the public in maintaining control measures. Nevertheless, R_o_ can indicate vital information to assist in planning public health interventions.

A lesser known measure of infectivity is the particle to plaque-forming unit ratio (P:PFU; Table 1). The P:PFU measures the fraction of viral particles able to infect susceptible cells in tissue culture under idealized in vitro conditions [9–11]. When P:PFU approaches 1, as occurs with bacteriophages, each viral particle is able to complete an infectious cycle in a susceptible cell (i.e., highly infectious to the cells in tissue culture) [9, 10]. For many animal viruses, the ratio is on the order of 500–10,000. There may be some uncertainty about this ratio since some viral particles used to infect cells in tissue culture may be nonviable [9, 11]. A high P:PFU ratio is often attributed to viral particles with incomplete genomes, structural capsid deficits, or lethal mutations [11]. The P:PFU ratio may add important insight into transmission dynamics of viral pathogens, especially when viral quantification is necessary [9, 12].

**Table 1:** Characteristics of viral transmission including the particle to plaque forming unit ratio assessed in cell culture and reproductive number assessed in epidemiologic studies

Virus	Particle:PFU	R_o_	R_o_/Particle:PFU	Transmission	Notes
Ebola *(-ssRNA)*	511 [12]	1.5–1.9 [21]	2.9 × 10^–3^–3.7 × 10^–3^	Bodily Fluids	Particle:PFU from strain at Walter Reed Medical Center [1]; R_o_ from 2014 epidemic [21]
Influenza A* *(-ssRNA)*	20–509	0.9–2.1 [6]	4.2 × 10^–2^–4.5 × 10^–2^	Predominantly Respiratory Droplet	Seasonal strains
Smallpox *(dsDNA)*	1–100 [9, 22]	6.87 [23]	1.46 × 10^–1^–14.6	Small particle aerosol	R_o_ from 1967 outbreak smallpox
VZV *(dsDNA)*	40000 [14]	10–12 [24]	2.5 × 10^–4^–3 × 10^–4^	Small particle aerosol; Vertical	R_o_ pre-vaccine
*Adenoviradae* *(dsDNA)*	20–100 [9]	2.34 [24]	2.3 × 10^–2^–1.2 × 10^–1^	Fecal–oral; Respiratory	
Rotavirus *(dsRNA)*	10 [9]	78.8 [26]	7.88	Fecal–oral; Droplet	R_o_ pre-vaccine estimation
HSV-1* *(dsDNA)*	50–200 [9]	2–5 [28]	2 × 10^–2^–4 × 10^–2^	Bodily Fluids, Sexual, Vertical	P:PFU antecedently recorded as 10:1
HSV-2 *(dsDNA)*	50–200 [9]	2.07 [29]	1 × 10^–2^–4 × 10^–2^	Sexual; Vertical	
Polio* *(*+ *ssRNA)*	36–1000 [9, 19]	5–6 [24]	6 × 10^–3^–1.4 × 10^–1^	Fecal–oral	
HPV *(dsDNA)*	10000 [9]	0.52–1.2 [30]	5.2 × 10^–5^–1.2 × 10^–4^	Sexual	STI strains; R_o_ assumes untreated population; ignores autoinnoculation
Coxsackie A *(*+ *ssRNA)*	210 [31]	2.5 [32]	1.2 × 10^–3^	Fecal–oral	
Measles* *(-ssRNA)*	10–200 [16, 17]	12–18 [3]	9 × 10^–2^–1.2	Small particle aerosol	R_o_ pre-vaccine
RSV (-*ssRNA)*	3200 [33]	1.2–3.0 [25, 34, 35]	3.8 × 10^–4^–9.4 × 10^–4^	Respiratory droplet; Fomite	
Mumps* *(-ssRNA)*	100–1000 [13]	10–12 [24]	1.2 × 10^–2^–1 × 10^–1^	Respiratory droplet	R_o_ pre-vaccine
SARS-CoV *(*+ *ssRNA)*	360 [36]	2.2–3.6 [4]	6.1 × 10^–4^–1 × 10^–2^	Respiratory droplet	Particle:PFU from gRNA
Rhinovirus* *(*+ *ssRNA)*	30–1000 [1, 18]	2–3 [35]	3 × 10^–3^–6.7 × 10^–2^	Respiratory droplet	Inferred P:PFU; R_o_ seasonal change
SARS-CoV-2 (+ *ssRNA)*	1000–1000000 [20, 37]	2.6–5.7 [27]	5.7 × 10^–6^–2.6 × 10^–3^	Respiratory droplet; possible aerosol	Early studies suggest R_o_ closer to 2.6

*VZV* varicella zoster virus, *HSV* herpes simplex virus, *HPV* human papillomavirus. Only “true aerosol” diseases were classified as aerosol. [24] Minor routes of transmission (i.e., fomite, vertical, animal) were ignored for graphical analysis

Multiple reference values for P:PFU ratios are from older literature that have not been revisited. Virologists often calculate P:PFU ratios for strains in their laboratories [13], but there are no standardized means of producing reference P:PFU ratios. Standardizing P:PFU ratio protocols and revisiting previously published data would be useful. For poliovirus, older sources document a P:PFU ratio ranging from 1000 to 30 and have not been revisited for since 1957 [9, 18, 19]. However, without a standardized means of collection, there is no way to assess which value is more accurate. Infective virions constituting 10% of a viral population vs. 0.5% of a population is a monumental difference which could have ramifications regarding transmission dynamics [12, 14, 15]. Furthermore, a lack of standardization may be associated with a wide range in P:PFU ratios for poliovirus, rhinovirus, and measles [9, 16–19]. In addition, P:PFU values must be interpreted with caution since viral passage in cell culture changes the P:PFU ratio as has been demonstrated for SARS-CoV-2 [20]. The inconsistency in cell line type (e.g., Vero 6 or HeLa cell lines) is another limitation due to lack of standardization [12]. Lastly, the P:PFU must be interpreted in context. A high P:PFU may represent defective interfering particles that have incomplete circular genomes and are unable to form plaques in culture but can still complete an infectious cycle in vivo by relying on complete helper genomes as reflected in one study in which high P:PFU strains of Ebola virus were still able to generate lethal infections [12].

We found inconsistencies among the dynamics of transmission for respiratory viruses. Influenza A virus has a lower P:PFU than respiratory syncytial virus (RSV), but RSV has a higher R_o_. This may reflect less than idealized tissue culture conditions for RSV or much more efficient person-to-person RSV transmission. SARS-CoV and rhinovirus have higher P:PFU than Influenza A virus but also higher R_o_. Perhaps this reflects the lack of ideal tissue culture conditions for SARS-Co-V and rhinovirus. With current limitations of P:PFU data, such discrepancies may be clarified when more tissue culture data are collected in a uniform manner.

Could an assessment of the R_o_/P:PFU ratio add to information garnered from either value alone as a virus such as SARS-CoV-2 evolves in its new human host? The new variants are evolving to more efficiently bind to ACE receptors on human cells [38] and this should lead to a lower P:PFU ratio, but it is unclear if or how this might affect person-to-person transmission of the virus, (i.e., affect the R_o_). If the R_o_/P:PFU ratio rises more quickly than the R_o_ alone, then it would suggest that improved receptor cell binding and/or cell entry did not translate into greater human-to-human transmission. Such comparisons may add insight as SARS-CoV-2 and other viruses adapt to a new host.

## Conclusion

As the COVID-19 pandemic continues the relationship between the P:PFU ratios and R_o_ may add to our understanding of SARS-CoV-2 as variants evolve to adapt to the new human host.

## Data Availability

Not applicable.

## Abbreviations

Ro: Basic reproductive number
P:PFU: Particle-to-plaque ratio
VZV: Varicella zoster virus
HSV: Herpes simplex virus
HPV: Human papillomavirus
